# Mechanism and Potential of Aqueous Enzymatic Extraction for Constructing Green Production System for Lipids and Proteins

**DOI:** 10.3390/foods14233981

**Published:** 2025-11-21

**Authors:** Zefang Jiang, Jiaqi Chen, Xin Guo, Fusheng Chen, Xingfeng Guo, Qiang Wang, Bo Jiao

**Affiliations:** 1 Key Laboratory of Agro-Products Processing, Institute of Food Science and Technology, Chinese Academy of Agricultural Sciences, Ministry of Agriculture and Rural Affairs, Beijing 100193, China; jiangzefang@caas.cn (Z.J.); chenjiaqi2970@163.com (J.C.); 2 College of Food Science and Engineering, Henan University of Technology, Zhengzhou 450001, China; guoxin@haut.edu.cn (X.G.); fushengc@haut.edu.cn (F.C.); guoxingfeng@haut.edu.cn (X.G.)

**Keywords:** aqueous enzymatic extraction, physical integration technology, cellular deconstruction, bioactive compounds, multiphase proteins

## Abstract

Conventional oil extraction methods face challenges such as nutrient loss, solvent residues, and protein denaturation. Aqueous enzymatic extraction (AEE), as a green alternative, offers mild processing and environmental benefits. However, its application is hindered by inefficient release of intracellular components due to rigid cell walls, difficulties in demulsifying stable oil–water interfaces, and insufficient valorization of by-products. Moreover, proteins are heterogeneously distributed among aqueous, emulsion, and solid phases with distinct functionalities, yet research remains disproportionately focused on aqueous-phase proteins, leading to suboptimal resource utilization. This study aims to elucidate targeted cell wall disruption mechanisms and the dynamic interplay between oil release and emulsion formation during enzymatic hydrolysis. By integrating physical-assisted technologies, we establish an oil–protein production system that overcomes efficient oil liberation and demulsification barriers. A multi-component functional evaluation framework is developed to systematically analysis oil nutritional properties and multi-phase protein functionalities. The proposed strategy of precision cellular deconstruction, technology integration, and component valorization provides a theoretical and technical foundation for enhancing AEE efficiency, producing high-quality oils, and advancing multi-phase protein functionalization.

## 1. Introduction

Vegetable oil, a primary global source of fats and oils, serves as both an essential component of human nutrition and a key industrial raw material for food, chemical, pharmaceutical, and energy sectors [1,2]. With growing global population and industrial demand, its strategic importance continues to rise. Over the past decade, worldwide vegetable oil consumption has maintained an average annual growth of 2.3%, with projections exceeding 220 million tons by 2025 [3]. Approximately 75% is directly used for edible purposes, providing essential fatty acids and fat-soluble vitamins crucial for infant development, chronic disease prevention, and nutritional balance [4,5].

Current production predominantly relies on two conventional methods: chemical solvent extraction and physical pressing. Solvent extraction using n-hexane achieves high oil yield but presents significant safety risks due to the solvent’s flammability and challenges in complete residue removal [6]. Subsequent high-temperature refining further degrades heat-sensitive bioactive compounds, substantially compromising nutritional quality [7]. While physical pressing avoids solvent residues, it faces limitations: hot pressing requires steaming and roasting pretreatment followed by high-pressure extraction, where elevated temperatures induce protein denaturation and Maillard reactions, reducing functional protein solubility in meal and increasing oil free radical content [8]. Although cold pressing preserves more bioactive components through lower temperatures, its oil yield remains 15–30% lower than chemical extraction, with high-fiber materials accelerating equipment wear and increasing production costs [9].

During the vegetable oil extraction process, the resulting oilseed meal, as a major by-product, is rich in high-quality protein. However, conventional protein extraction methods, such as alkaline solution and acid precipitation and organic solvent extraction, often lead to protein denaturation, impaired functional properties, or pose risks of solvent residues in the meal during recovery [10]. These limitations make it difficult to utilize the protein for high-value applications, largely using to animal feed or fertilizer, thereby resulting in a significant waste of protein resources [11]. Consequently, the development of green processing technologies capable of simultaneously and efficiently extracting high-quality oil and protein is crucial for enhancing the comprehensive economic value of oilseed crops and achieving sustainable development [12].

To address the aforementioned challenges, building an efficient, low carbon, and co-production green extraction system for vegetable oils has become an industry consensus. Both academic and industrial research have shifted focus toward green processes such as aqueous enzymatic extraction (AEE) and supercritical CO_2_ extraction [13,14]. In addition, the technology for aqueous extracting vegetable oil in the form of intact oleosomes constitutes another important research direction in the current field of oilseed processing. These technologies aim to overcome the limitations of traditional methods, achieving efficient extraction of oils and proteins, thereby advancing oil and grain processing from a single-product focus to multi-component high-value utilization. Among these, aqueous enzymatic extraction technology demonstrates unique advantages [15]. This method is primarily applied to oil-bearing crops such as soybeans, peanuts, and sunflower seeds, as well as cereals and their processing by-products. As a green technique, it uses water as the medium instead of organic solvents, operates under mild conditions, and completely eliminates risks of flammability, explosiveness, and solvent residues. Compared to water extraction, AEE significantly enhances the disruption efficiency of plant cell walls and lipoprotein complexes through the addition of specific enzyme preparations, thereby overcoming the limitations of conventional water extraction, namely low extraction yield and the difficulty in releasing intracellular contents [16]. In contrast to supercritical CO_2_ extraction technology, the advantage of AEE lies in its ability to simultaneously recover high-quality protein, achieving a holistic utilization of oilseed crops rather than separately extracting oil and protein [17].

AEE employs biocatalytic regulation for targeted multi-component separation, offering a sustainable pathway for oil and grain processing [18]. However, its industrialization faces bottlenecks including lower oil yields than chemical extraction, difficulties in demulsifying stable emulsions, and inadequate valorization of by-products. To overcome the aforementioned bottlenecks, this paper proposes an innovative research pathway centered on the core architecture of precise cellular dissociation, efficient technology integration, targeted component valorization. By deeply analyzing the structure of oilseed crop cells, we reveal the targeted disruption mechanism of AEE and elucidate the dynamic interplay between oil release and emulsion formation during enzymatic hydrolysis. Through the innovative integration of assisted physical technologies, we construct a novel oil-protein green production system, thereby overcoming technical barriers in efficient oil liberation and demulsification. Furthermore, based on multicomponent functional profiling, we systematically establish a synergistic evaluation framework for assessing the nutritional properties of oils and functional characteristics of multiphase proteins. This integrated approach provides fundamental theoretical support for enhancing extraction efficiency and amplifying industrial application and economic viability of AEE technology.

## 2. Cell Structure of Oilseed Crops

**Figure 1:**
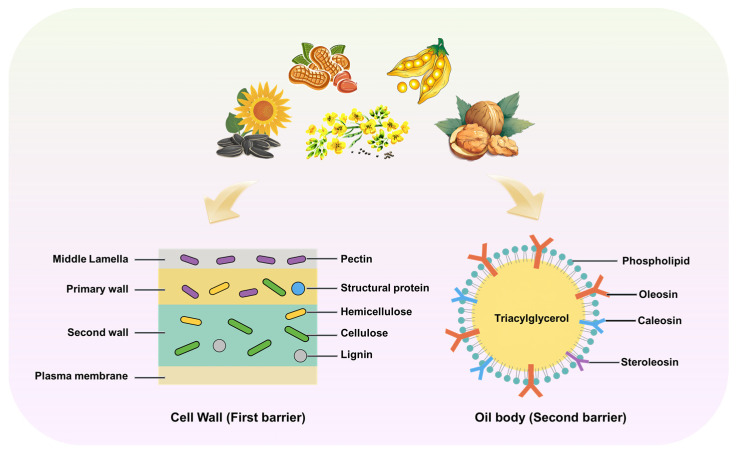
Schematic diagram of oilseed crop cell structure.

Vegetable oils are primarily stored in the oil bodies of oilseed crop cells. The core of oil bodies consists of triacylglycerols, enclosed by a phospholipid–protein bilayer membrane, and embedded within the cell wall network composed of cellulose, hemicellulose, and pectin (Figure 1). Oils often form stable lipoprotein complexes with storage proteins and polysaccharides through hydrophobic interactions, hydrogen bonds, or covalent bonds [19]. This multi-layered spatial structure significantly impedes the efficient release of oil. Thus, a thorough understanding of the cellular structure of oilseed crops is crucial for elucidating the mechanisms of both oil extraction and enzymatic action.

### 2.1. Cell Wall

The plant cell wall is a structure composed of multiple complex macromolecules, with structural polysaccharides constituting its core components [20]. Cellulose serves as the primary skeletal material, formed by long chains of glucose molecules tightly linked via specific glycosidic bonds into robust microfibrils. Hemicellulose, a class of highly branched heteropolysaccharides containing various monosaccharides such as xylose, mannose, and arabinose, intertwines around the cellulose, fulfilling roles of connection and space-filling. Pectin, rich in galacturonic acid, is predominantly located in the intercellular spaces and primary walls, acting as an adhesive that bonds adjacent cells. Additionally, structural proteins rich in specific amino acids enhance wall flexibility through intermolecular cross-linking, while the hydrophobic lignin polymer extensively deposits in the secondary walls, forming a sturdy and degradation-resistant complex with hemicellulose.

The cell wall is not homogeneous but exhibits a sophisticated layered and networked architecture. In plant cells, the outermost layer is the middle lamella, primarily composed of pectin, which tightly cements adjacent cells. Immediately interior to this lies the more flexible primary wall, where cellulose microfibrils are embedded like steel rebars within a gel-like matrix of hemicellulose, pectin, and structural proteins. Many cells further develop a thicker and denser secondary wall toward their interior. Within this secondary wall, cellulose microfibrils adopt a more tightly packed and ordered arrangement, extensively cross-linked with substantial amounts of hemicellulose and hydrophobic lignin [21]. This multiscale, heterogeneous architecture forms the foundation of its exceptional mechanical strength and resistance to penetration.

The composition and structure of the cell wall collectively establish both physical and chemical barriers for the aqueous enzymatic extraction of intracellular substances [22]. Physically, the densely interwoven polysaccharide network forms small pores, acting like a molecular sieve that severely impedes the diffusion and access of large enzyme molecules to target substrates. The tightly packed polymers generate significant steric hindrance, restricting effective contact between enzyme molecules and their specific binding sites. Hydrophobic lignin regions and the surface cuticular wax layer further repel the approach of water-soluble enzymes. Chemically, covalent cross-links formed between components, particularly the connections between lignin and carbohydrates, and the cross-linking of structural proteins, enhance the overall rigidity and stability of the network, resisting enzymatic degradation [23]. Additionally, the highly ordered crystalline regions of cellulose molecules are inherently recalcitrant to enzymatic hydrolysis. Consequently, the efficacy of aqueous enzymatic extraction is highly dependent on effectively overcoming this barrier. This necessitates the precise selection and formulation of tailored enzyme cocktails based on the dominant components of the target raw material cell wall.

### 2.2. Oil Body

Oil bodies are specialized subcellular organelles in plant cells dedicated to triacylglycerol (TAG) storage, abundantly present in oilseed crops such as rapeseed, soybean, peanut, sunflower, and oil palm mesocarp [24]. Structurally, TAG constitutes the core component of oil bodies, existing as liquid droplets whose hydrophobic nature establishes a primary physical barrier. During seed dehydration and maturation, TAG molecules undergo hydrophobic interaction-driven polymerization and phase separation, forming a homogeneous oil phase 0.5–2 μm in diameter. This hydrophobic environment impedes the diffusion of water-soluble enzymes, preventing their access to the lipid–water interface. During extraction, liberated TAG readily coalesces via hydrophobic interactions, complicating subsequent separation [25]. In AEE, disrupting the peripheral membrane structure is essential to expose TAG, while surfactants are employed to reduce interfacial tension and facilitate emulsified release.

The phospholipid monolayer membrane, composed primarily of phosphatidylcholine and phosphatidylethanolamine, forms a critical interfacial barrier through its amphipathic molecular arrangement. The hydrophilic headgroups orient toward the aqueous cytosol, while the hydrophobic tails insert into the TAG core, establishing a thermodynamically stable interface. Negatively charged headgroups (e.g., phosphate groups in phosphatidylcholine) generate a high surface potential, preventing oil body coalescence via electrostatic repulsion. This phospholipid layer further provides an insertion matrix for the hydrophobic domains of oil body membrane proteins, with both components synergistically enhancing membrane rigidity [26]. Phospholipases can hydrolyze either phospholipid headgroups or fatty acid chains, thereby disrupting membrane integrity, releasing lysophospholipids, and ultimately triggering oil droplet coalescence.

Oleosins constitute over 90% of oil body membrane proteins. Their centrally conserved proline knot structure penetrates the phospholipid monolayer and embeds into the TAG phase, forming a hydrophobic anchor domain. The N- and C-terminal amphipathic α-helices extend into the cytosol, creating a ~10 nm charged polypeptide layer that impedes protease access to the membrane surface through combined steric hindrance and electrostatic repulsion. The N-terminal proline-rich motif adopts a rigid conformation resistant to proteolytic cleavage, while the folded C-terminal domain shields enzymatic recognition sites [27]. During AEE, alkaline proteases must be prioritized to hydrolyze the hydrophilic termini of oleosins, thereby dismantling the steric barrier and exposing the phospholipid layer for subsequent degradation.

While the natural stability of oil bodies is essential for lipid storage in plant seeds, it poses a fundamental obstacle to industrial oil extraction. A dual-barrier system impedes effective TAG release: primary physical confinement by cell walls/membranes, and secondary interfacial isolation via the phospholipid monolayer and dense oleosin protein layer. In solvent extraction, oleosin hydrophilic shell impedes solvent penetration. During mechanical pressing, although high-pressure shear forces disrupt oil body structures, the liberated TAG combines with intracellular water, proteins, and phospholipids to form highly viscous water-in-oil (W/O) emulsions [28]. This substantially increases system viscosity, obstructs oil outflow, and elevates residual oil rates. More critically, phospholipids and oleosins released from disintegrated oil bodies act as potent surfactants that rapidly adsorb at oil–water interfaces, forming stable emulsifying films that encapsulate lipid droplets within the aqueous phase.

## 3. Aqueous Enzymatic Extraction Technology

Enzymatic extraction is a green processing technology based on biocatalysis, with its complete technological process comprising three interconnected stages. In the raw material pretreatment stage, physical methods such as mechanical crushing are employed to effectively disrupt the cell wall structure of oilseed crops, thereby increasing the contact area between enzymes and substrates [29]. Upon entering the enzymatic hydrolysis stage, specific enzymes such as cell wall-degrading enzymes or proteases are precisely selected according to the characteristics of the target products, and controlled hydrolysis is conducted under optimal temperature and pH conditions to simultaneously release oils and proteins [17,30]. In the final component separation stage, a multiphase system consisting of oil, emulsion, protein solution, and residue is obtained through centrifugal separation. This is combined with refined separation techniques such as enzymatic demulsification and isoelectric point precipitation to ultimately achieve efficient separation and recovery of oils and proteins [31]. The entire process is carried out in a mild aqueous environment, fully leveraging the specific catalytic action of enzymes, thereby laying a solid foundation for constructing a green and efficient aqueous enzymatic extraction system. As shown in Table 1, in the enzymatic extraction process, parameters such as enzyme type, enzyme concentration, reaction time are the main process parameters that collectively determine the yield and quality of the final products.

### 3.1. Key Process Parameters in AEE

#### 3.1.1. Enzyme Type

The choice of enzyme type is the foundation of the aqueous enzymatic process, as it determines the targeting and specificity of the hydrolysis, directly affecting the yield and quality of the target products (oil or protein). Different enzyme preparations act on distinct components of the cellular structure, necessitating precise matching based on both the raw material characteristics and the extraction objectives [33]. For instance, for oil-bearing crops with dense cell wall structures, such as rice bran and algae, cell wall-degrading enzymes like pectinase, cellulase, and hemicellulase are the preferred choices. These enzymes work synergistically to disrupt the cell wall’s network structure and break down the physical barrier, thereby releasing the intracellular contents more efficiently [42]. Mounika et al. reported that in the aqueous enzymatic extraction of rice bran oil, the combination of cellulase, amylase, and protease significantly improved the oil yield compared with single-enzyme treatments, demonstrating a clear synergistic effect of multiple enzymes [45]. Conversely, when the objective leans towards obtaining functional proteins or peptides, proteases become central. They directly hydrolyze storage proteins and membrane proteins, breaking down large molecular proteins into soluble peptides and amino acids [46]. However, it is crucial to control the degree of hydrolysis to prevent the production of bitter peptides. The recent study by Lolli et al. demonstrated that a one-pot enzymatic extraction approach can effectively recover both oil and protein from fruit seeds and kernels, highlighting the potential of protease-based systems for dual-target extraction processes [17].

#### 3.1.2. Enzyme Concentration

Enzyme concentration is a key parameter affecting both reaction kinetics and economic feasibility. It directly determines the collision frequency between enzyme and substrate molecules, thereby influencing the hydrolysis rate and extent. Generally, within a certain range, the yields of oil and protein increase significantly with rising enzyme concentration. However, once the enzyme concentration exceeds a critical threshold, the growth in yield plateaus or even declines due to substrate saturation or the emergence of product inhibition effects. Islam et al. optimized the enzymatic hydrolysis of soybean protein using Alcalase and found that increasing the enzyme-to-substrate ratio from 1 to 2.5% (*w*/*w*, based on protein) significantly enhanced the degree of hydrolysis, whereas further increases to 3.0–3.5% did not lead to any significant improvement, indicating an apparent saturation of the reaction rate at high enzyme concentrations [47]. Excessively high enzyme concentrations not only lead to wasteful use of enzymes and increased costs but may also cause excessive protein hydrolysis, generating an abundance of small bitter peptides that ultimately impair the product’s flavor and certain functional properties, such as emulsification capacity.

#### 3.1.3. Reaction Time

Reaction time acts synergistically with enzyme concentration, collectively determining the progression and endpoint of the hydrolysis reaction. An optimal reaction duration should ensure sufficient interaction between the enzyme and substrate to achieve the desired degree of hydrolysis. If the time is too short, the reaction remains incomplete, leading to low extraction yields; conversely, excessively prolonged times may result in secondary degradation of released products, microbial contamination, or unnecessary energy consumption. Meng et al. investigated the enzymatic hydrolysis of peanut protein using papain to reduce allergenicity and improve functional properties [48]. They observed that moderate hydrolysis markedly enhanced the solubility and foaming capacity of peanut protein, indicating that a controlled reaction time favors the exposure of hydrophilic groups and the generation of peptides with desirable interfacial activity. However, when hydrolysis was extended excessively, the emulsifying stability declined, which was attributed to over-degradation of large molecular peptides that are essential for maintaining a stable emulsion interface.

### 3.2. Physical Integration AEE Technology

**Figure 2:**
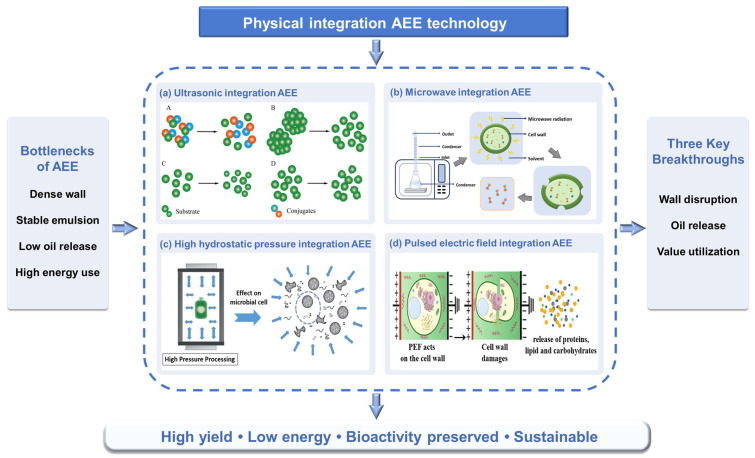
Physical integration strategies in aqueous enzymatic extraction (AEE). (**a**) Ultrasonic integration AEE: cavitation and shear effects induce substrate disruption and structural rearrangement, shown as (**A**) dispersed substrates, (**B**) particle aggregation, (**C**) cavitation-driven dispersion, and (**D**) stabilized conjugates [50]. (**b**) Microwave-assisted aqueous enzymatic extraction (AEE). Microwave radiation induces cell wall disruption by internal pressure, facilitating protein release into the solvent [51]. (**c**) Ultra-high pressure integration AEE: pressure-induced structural modifications increase cell permeability and enzyme accessibility [52]. (**d**) Pulsed electric field integration AEE: electroporation and localized disruption facilitate emulsion breakdown and bioactive release [53].

AEE stands as a green and safe technology for oil extraction. However, its industrial-scale application faces dual bottlenecks: Firstly, the dense cellulose–hemicellulose composite cell wall structure of oilseeds is difficult to efficiently disrupt solely via enzymatic hydrolysis [49]. Secondly, the enzymatic process readily forms highly stable oil–water emulsions, leading to a significant reduction in oil recovery yield. The introduction of physical assistance is critically essential to overcome these bottlenecks. By integrating mechanical forces and field energy provided by physical means such as ultrasound, microwave, ultra-high pressure, or pulsed electric fields, this approach effectively disrupts dense cell wall structures, significantly reducing the resistance to enzymatic hydrolysis and lowering enzyme requirements. Simultaneously, it destabilizes stable emulsion systems through disruption of the oil–water interfacial film, leading to a substantial increase in free oil yield (Figure 2). This review focuses on the synergistic enhancement and green upgrading of AEE through physical assistance. By collaboratively optimizing mass transfer efficiency, effectively suppressing emulsification effects, and reducing overall production costs.

#### 3.2.1. Ultrasonic Integration AEE

Ultrasonic integration aqueous enzymatic extraction (UAEE) technology, which couples the physical effects of ultrasonic fields with the biocatalytic action of enzymatic hydrolysis, significantly enhances the extraction efficiency and quality of oils and bioactive compounds, positioning it as a prominent research focus in green extraction [50]. This synergistic enhancement operates through three interconnected mechanisms: Firstly, ultrasound-induced cavitation in liquid media generates localized high temperature, high pressure, and intense shear forces during microbubble collapse, efficiently disrupting the dense cellulose–hemicellulose composite structure of oilseed cell walls. Secondly, cavitational shockwaves intensify mass transfer by accelerating enzyme diffusion toward substrates while simultaneously reducing oil droplet size, thereby expanding oil–water interfacial contact area and elevating effective substrate concentration. Finally, optimized enzymatic processing is achieved through bioeffects where appropriate ultrasound intensity induces enzyme conformational changes to expose active sites, while concurrently modifying substrate structures via exposed hydrophobic protein groups and dissociated lipoprotein complexes, accelerating enzyme–substrate binding kinetics.

UAEE has gained widespread adoption across food, pharmaceutical, and biochemical sectors, while this section specifically targets its applications in agricultural processing. Regarding vegetable oils and protein extraction, neutrase-assisted extraction coupled with 21 min ultrasound pretreatment for *Cinnamomum camphora* seeds achieved an 88.65% oil yield under optimized conditions (solid-to-liquid ratio 1:7 *w*/*v*, 4.5 h hydrolysis), representing a 25% increase over conventional enzymatic methods [54]. Concurrently, ultrasound-assisted papain hydrolysis of defatted *Pinus pumila* nut meal (40 kHz, 200 W, 30 min) achieved an 83.72% protein extraction yield, accompanied by markedly improved particle morphology and interfacial properties conducive to emulsification [55]. Additionally, in valorizing grain byproducts, soybean residue treated with 300 W ultrasound coupled with dual-enzyme (Alcalase Flavourzyme) hydrolysis attained 52.07% degree of hydrolysis, yielding peptides with superior ACE inhibitory activity [56]. Furthermore, for bioactive compound extraction from *Fructus aurantii*, an ultrasound-enzyme assisted ethanol/ammonium sulfate aqueous two-phase system incorporating neutrase-pectinase (1:1) achieved 92.27 mg/g neohesperidin yield alongside 30% impurity reduction—demonstrating viability for high-purity nutraceutical production [57].

#### 3.2.2. Microwave Integration AEE

Microwave integration aqueous enzymatic extraction (MAEE) technology, which synergistically combines microwave-induced thermal and non-thermal effects with enzyme-specific biocatalysis, has emerged as a significant research direction in green manufacturing [58]. Microwaves cause polar molecules within the material to oscillate at high frequencies, generating instantaneous high temperatures. This thermal effect leads to the vaporization of internal cellular moisture, creating steam pressure that ruptures the cellulose–hemicellulose complex structure of the cell walls, promoting the release of oils or active components [59]. The microwave electric field alters the arrangement of polar side chains in enzyme proteins, reducing the reaction activation energy and accelerating substrate–enzyme binding. For example, in the immobilization of thermophilic bacterial protease, 40W microwave radiation induced conformational extension of the enzyme. This increased the loading rate to 93.2% and boosted catalytic activity to 1.6 times that of the free enzyme, while reducing the immobilization time from 20 h to just 3 min [60]. Concurrently, microwaves can also inhibit emulsification. As demonstrated in okara protein extraction, microwave pretreatment lowered the oil–water interfacial tension, increasing the free oil yield to over 90% [61].

MAEE technology has currently been applied in the food, pharmaceutical, and biochemical industries. Using microwave pretreatment combined with complex enzymatic hydrolysis (cellulase 1.5% + pectinase 2.0% + protease 0.25%) at pH 4.5 and 45 °C for 6 h, an oil yield of 27.9% was achieved—approximately 20% higher than traditional enzymatic methods. The non-thermal effect of microwaves modifies fiber structure, increases porosity, and enhances antioxidant activity [62]. When applied to plantain pulp and peel via microwave-high temperature cooking-assisted enzymatic treatment, the soluble dietary fiber (SDF) yield reached 8.28% and 5.46%, respectively, with water-holding capacity increasing from 2.93 g/g to 3.90 g/g [63]. For high-value utilization of byproducts, continuous microwave-assisted dual-enzyme (alkaline protease flavourzyme) hydrolysis of cottonseed meal comprehensively improved the functional properties of hydrolysates [64]. Microwave radiation exposes hydrophobic groups of proteins, enhancing interfacial activity and making it suitable for replacing synthetic food additives.

#### 3.2.3. High Hydrostatic Pressure Integration AEE

High hydrostatic pressure integration aqueous enzymatic extraction (HAEE) technology, which synergistically combines high-pressure physical effects with enzymatic biocatalysis, has emerged as a pivotal innovation in the field of green manufacturing. High hydrostatic pressure treatment ruptures cell walls through pressure effects, disrupting the cellulose and hemicellulose complex structure and increasing substrate–enzyme contact area [65]. Simultaneously, high pressure alters the physical properties of extraction solvents, accelerating the solubilization rate of polar components. Notably, high hydrostatic pressure exerts a biphasic effect on enzyme activity: Moderate pressure (≤500 MPa) extends enzyme conformation, exposing active sites and enhancing enzymatic activity. Pressures >500 MPa disrupt enzyme spatial structures, triggering a deactivation effect.

HAEE technology has currently achieved innovative applications in multiple fields. 300 MPa high hydrostatic pressure assisted enzymatic hydrolysis increased the brightness of sunflower meal protein by 46% and improved in vitro digestibility to 96.21%. High pressure induced the proportion of protein β-sheets to increase to 42.63%, enhancing structural flexibility and reducing color darkening caused by chlorogenic acid oxidation [66]. In DHA algal oil extraction from *Schizochytrium* sp., high pressure homogenization combined with alkaline protease hydrolysis doubled cell wall disruption efficiency, achieving an oil extraction rate of 92.12%—a 12.12% increase compared with single high-pressure methods [67]. This technology breaks through the dense barrier of microalgal cell walls and significantly reduces energy consumption. During antioxidant peptide preparation from *Pelophylax nigromaculatus* skin, high hydrostatic pressure-assisted dual-enzyme hydrolysis yielded 25.26% low molecular weight peptides (1–2000 Da), with DPPH radical scavenging rate of 76.93% and ABTS scavenging rate of 59.23% [68]. Enhanced antioxidant activity was verified in *C. elegans* models, providing pathways for high value utilization of aquatic byproducts.

#### 3.2.4. Pulsed Electric Field Integration AEE

Pulsed electric field integration aqueous enzymatic extraction (PAEE) technology operates by combining the physical effects of high-voltage pulsed electric fields with enzymatic biocatalysis. Its core mechanism relies on the synergistic interaction between electroporation effects and enzymatic activity [69]. The pulsed electric field polarizes phospholipid molecules in cell membranes within microseconds, forming reversible or irreversible nanoscale pores, substantially increasing membrane permeability. Simultaneously, the pulsed electric field exerts a biphasic effect on enzymes: moderate electric fields (10–20 kV/cm) cause conformational extension of enzyme proteins, exposing active sites and enhancing enzyme activity; high-intensity electric fields (>30 kV/cm) disrupt enzyme spatial structures and can terminate enzymatic reactions. Important applications in aqueous enzymatic extraction are highlighted through the pulsed electric field disruption of oil–water interfacial film stability via charge redistribution, which inhibits emulsification [70]. During oil extraction, secondary pulsed electric field treatment induces polarization-induced collision of emulsion droplets, achieving demulsification efficiency >95% with only 1/10 the energy consumption of thermal demulsification [71].

In the development of emerging protein resources, physical integrated extraction technology plays a critical role and serves as a core approach for efficient protein extraction and preparation. Efraim et al., have developed a novel continuous pulsed electric field device combined with enzymatic treatment and spray drying for the aqueous fractionation of *Ulva* sp. protein. This process achieved a protein extraction yield of 8.79 ± 0.58% (*w*/*w*). These findings highlight the potential of this integrated approach for efficiently obtaining high-quality protein ingredients from green marine macroalgae [72]. Regarding high-value utilization of byproducts, after pulsed electric field pretreatment (5 kV/cm, 120 pulses) followed by alkaline solution extraction, although extraction yield wasn’t significantly improved, brewer’s spent grain protein exhibited an 81% increase in solubility, a 104% improvement in foaming capacity, and water-holding capacity rising from 3.9 g/g to 11.5 g/g [73].

#### 3.2.5. Comparison of Physical Technologies Integrated with AEE

The comparative analysis (Table 2) indicates that no single technology—ultrasound, microwave, high hydrostatic pressure, or pulsed electric field—is universally superior. The selection of an auxiliary method is contingent on a trade-off specific to the oilseed material and the desired characteristics of the products (oil and protein). For instance, Ultrasonication-induced cavitation can compromise product quality by denaturing proteins and oxidizing lipids [74]. Furthermore, its scale-up is hindered by uneven energy distribution in industrial reactors, leading to inconsistent results and high operational costs. While microwave offers the highest extraction efficiency, its thermal intensity may compromise the functional properties of the resulting proteins, making it less suitable for high-value protein co-production [75]. Conversely, pulsed electric field provides a mild, non-thermal alternative ideal for preserving protein native structures, yet its application is currently constrained to liquid or semi-solid matrices, posing a challenge for some oilseeds [76]. Despite its gentle and efficient extraction, the industrial adoption of high hydrostatic pressure is severely hampered by prohibitively high equipment costs and a batch-processing nature that limits throughput [77].

The selection of physical integration technologies should be based on specific Production objectives. If the goal is to preserve the native activity of proteins or prevent lipid oxidation, ultra-high pressure and pulsed electric fields are preferable choices. For heat-insensitive applications, microwave and ultrasonic technologies offer greater speed advantages. Ultrasonic and microwave techniques involve relatively lower equipment and operational costs, while pulsed electric fields and microwaves show greater potential for continuous production. Future trends will focus on combining these physical technologies or achieving more precise temporal coupling with enzymatic hydrolysis processes to strike a balance between efficiency and quality [78].

**Table 2:** A Comparative Analysis of physical technologies integrated with AEE.

Technology	Efficiency (Typical Oil Yield Improvement)	Key Advantages	Critical Limitations	Reference
Ultrasound	10–25%	Effective for hard-walled materials; rapid	High energy consumption; potential for protein/peptide degradation; probe erosion at scale	[79,80,81,82]
Microwave	15–30%	Rapid, uniform heating; high efficiency	High energy consumption; potential for protein/peptide degradation; probe erosion at scale	[83,84,85,86]
High hydrostatic pressure	5–20%	Excellent for preserving thermo-labile compounds	Extremely high capital cost; batch processing limits throughput	[87,88,89]
Pulsed Electric Field	10–25%	Low thermal load, energy-efficient for liquids	Limited efficacy on dry or high-fat materials; electrode fouling	[90,91,92]

### 3.3. Economic and Environmental Benefits

The transition of aqueous enzymatic extraction (AEE) from laboratory success to industrial-scale production faces significant challenges in process integration and scale-up. The technology typically involves multiple unit operations—including raw material pretreatment, enzymatic hydrolysis, demulsification, centrifugal separation, and drying—each of which critically influences overall economic viability when scaled. Enhancing efficiency through the development of high-performance reactors and integrated hybrid systems that combine physical assistance technologies with in-line enzymatic processes represents a key research direction for achieving scalability and reducing both time and energy consumption per unit product [93]. Furthermore, a life cycle assessment (LCA) provides a quantitative foundation for transitioning this technology from a conceptual stage to sustainable commercialization.

#### 3.3.1. Enzyme Recycling Strategies

AEE demonstrates significant potential for the extraction of oils and proteins, yet its industrialization faces challenges related to the cost of enzyme preparations. The use of high-purity commercial enzymes entails substantial expenses, which is one of the key factors hindering the cost competitiveness of AEE compared to conventional extraction technologies. Therefore, strategies for enzyme recovery and reuse are critical to the economic feasibility of this technology.

Among the various recycling strategies, immobilized enzyme technology is regarded as the most promising solution. This approach involves attaching enzymes to specific insoluble carriers through physical or chemical methods, enabling their repeated use. The technology offers advantages such as reusability, enhanced stability, and ease of separation [94]. In addition to enzyme immobilization, other recycling strategies such as ultrafiltration membrane recovery and aqueous two-phase system separation can also be applied to recover free enzymes [95]. Ultrafiltration separates enzymes from hydrolysates based on molecular size, though its application is often constrained by membrane fouling and flux decline. Aqueous two-phase systems leverage differential partitioning of enzymes and products between phases for separation, yet their cost and recovery efficiency still require further optimization. Future research should prioritize the development of efficient, low-cost enzyme immobilization techniques or innovative recovery processes. Without breakthroughs in this critical aspect, the high cost of enzymes will remain a major obstacle to the widespread industrial adoption of AEE and its ability to compete with traditional methods.

#### 3.3.2. Life Cycle Assessment (LCA)

LCA serves as a systematic methodology for evaluating the sustainability of AEE. It quantifies the environmental impacts associated with a product or process throughout its entire life cycle providing a scientific basis for assessing key sustainability indicators [96]. By integrating energy use, emissions, resource depletion and human-health-related endpoints in a single framework, LCA also enables a direct comparison between AEE and conventional technologies such as solvent extraction or mechanical pressing, thus guiding process optimization and technology selection.

In terms of resource consumption and human toxicity impacts, AEE demonstrates distinct advantages by completely avoiding the use of fossil-derived n-hexane. Cravotto et al. reviewed the use of technical hexane in the food industry and pointed out that n-hexane, a petroleum-derived solvent widely employed for vegetable oil extraction, is neurotoxic and has been recognised as a cause of occupational diseases in several European countries, raising concerns about worker safety and the continued reliance on fossil-based resources [97]. By comparison, AEE completely eliminates this environmental concern. From a regulatory perspective, solvent-free AEE is also consistent with current food-safety frameworks, as both European and United States authorities (e.g., EU Directive 2009/32/EC and US FDA regulations on extraction solvents) impose strict limits on the use and residual levels of petroleum-derived extraction solvents in edible oils and protein ingredients. By replacing n-hexane with water as the extraction medium, AEE-derived products inherently avoid solvent-residue issues and thus facilitate regulatory compliance and clean-label positioning. The advantages of AEE are particularly evident regarding volatile organic compound (VOC) emissions. A comparative analysis by Usman et al. found that traditional solvent-based oil extraction processes relying on volatile organic solvents like n-hexane can lead to significant VOC emissions across the production chain, which in turn have the potential to contribute to photochemical ozone and smog formation [98]. In contrast, the AEE pathway produces virtually no such atmospheric pollutants, significantly reducing its potential impact on regional air quality.

Most importantly, LCA captures the circular economy value of AEE achieved through holistic biomass utilization. According to an LCA study by Bashiri et al. (2024), the integration of enzymatic hydrolysis with comprehensive biomass valorization enables substantial reductions in environmental burden [99]. By recovering high-value co-products such as proteins, oils, and carbohydrates, the overall process achieves improved resource efficiency and markedly offsets impact in categories such as eutrophication and acidification. This model enables AEE to exhibit superior environmental performance compared to single-output systems in multiple impact categories, including eutrophication potential and acidification potential, thereby providing a scientific foundation for establishing a more sustainable oil processing system.

## 4. High Quality Oils

**Figure 3:**
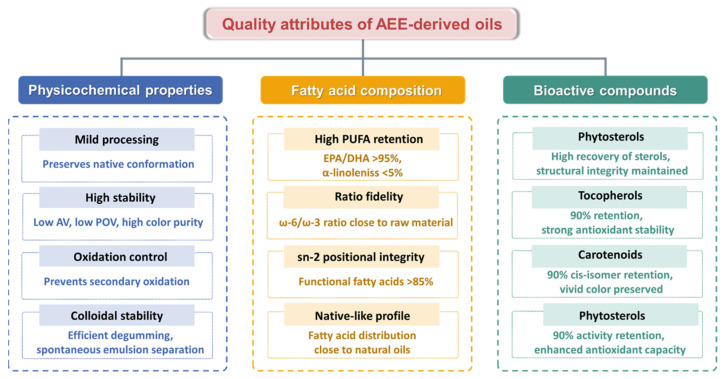
Quality characteristics of oils from AEE.

AEE as a green biomanufacturing technology, achieves efficient lipid release and quality enhancement through the integrated action of biocatalytic hydrolysis and mild physical fields. The resulting lipids exhibit fundamental distinctions from those obtained via traditional pressing or organic solvent extraction (Figure 3). The gentle non-thermal processing mechanism maximally preserves lipids native conformation, demonstrating exceptional stability through low acid value (AV), low peroxide value (POV), and high color purity. The targeted selectivity of enzymatic hydrolysis effectively prevents oxidative degradation of unsaturated fatty acids [100]. This significantly enhances the retention of thermally sensitive functional factors (e.g., oleic acid, α-linolenic acid), with fatty acid ratios closer to native profiles. The cell-level disruption and release mechanism substantially enriches phytosterols, tocopherols, carotenoids, and polyphenols while maintaining the integrity of these natural compounds [101]. This marked quality elevation establishes AEE derived oils as ideal feedstocks for the health food and premium pharmaceutical sectors.

### 4.1. Physicochemical Properties

AEE achieves oil release at low temperatures through the synergistic action of biological enzymes targeting cell wall degradation and mild physical fields, fundamentally avoiding the thermal degradation inherent in conventional processes. Compared to pressing methods where Maillard-induced browning causes color deterioration, aqueous enzymatic extraction preserves the oil’s natural color avoiding high-temperature thermal degradation, while maintaining favorable rheological properties that support ease of processing [15]. In contrast to solvent extraction requiring 110 °C hexane recovery that triggers flavor loss, aqueous enzymatic extraction eliminates solvent residues and fully retains low-boiling-point flavor compounds, achieving simultaneous enhancement of color and rheological properties.

In terms of oxidative stability, aqueous enzymatic extraction establishes a defense system featuring precise enzyme regulation and oxidative chain reaction interruption. The mild enzymatic environment inhibits lipase activation, stabilizing the acid value at 0.8–1.2 mg KOH/g-60% lower than traditional pressed oils [102]. Pulsed electric field-assisted instant enzyme inactivation blocks lipoxygenase catalytic pathways, controlling peroxide value at 2.1–4.5 meq O_2_/kg, significantly below conventional methods. Cell-level disruption avoids metal ion release, reducing secondary oxidation products by 80%, with anisidine value dropping to 4.3–7.8 versus 12.6–22.4 in traditional oils [103].

Colloidal stability improvement stems from synergistic control of phospholipids and emulsifying factors. Phospholipase A1 targeted hydrolysis transforms non-hydratable phospholipids into hydratable forms, achieving post-degumming phospholipid residues <10 mg/kg, 95% lower than conventional hydration degumming. Pulsed electric field demulsification adjusts droplet zeta potential from −35 mV to −8 mV, enabling spontaneous oil–water separation without centrifugal aids [104]. Cryo-electron microscopy further reveals intact lipoprotein complexes conferring Pickering stabilization, revolutionizing the traditional multi-stage refining model.

### 4.2. Fatty Acid Composition

The AEE technology preserves the natural conformation and compositional balance of fatty acids at the molecular level to the greatest extent. Oils extracted by this method demonstrate significant superiority over traditional processes in terms of fatty acid profile integrity and proportional fidelity [42]. Firstly, this method significantly enhances the retention rate of polyunsaturated fatty acid (PUFA). EPA/DHA retention exceeds >95%, compared to 70–80% for solvent extraction. α-Linolenic acid loss is maintained at <5%. In contrast, traditional processes induce oxidation and polymerization reactions due to high temperatures/solvents, resulting in PUFA losses of 20–30% [105]. Secondly, the aqueous enzymatic method precisely maintains the natural fatty acid ratios. The ω-6/ω-3 ratio strictly adheres to the raw material’s initial state (e.g., perilla oil at 0.3:1), with fluctuations controlled within ±0.05. Conversely, conventional refining processes cause systematic imbalance in this ratio due to the selective degradation of ω-3 fatty acids [106]. Lastly, this method fully preserves the sn-2 positional structure of triglycerides. Functional fatty acids (e.g., sn-2 palmitic acid) account for >85%, achieving structural similarity to breast milk lipid configuration. In contrast, traditional processes disrupt the spatial conformation at the sn-2 position through random esterification reactions, reducing this proportion to <40% [107].

### 4.3. Bioactive Compounds

AEE stands as a pivotal green technology for the efficient and high-fidelity recovery of critical bioactive compounds, notably tocopherols, polyphenols, phytosterols, and carotenoids from plant matrices [15,86]. Operating under mild, aqueous conditions without organic solvents, AEE fundamentally circumvents the thermal degradation and chemical alterations inherent in conventional extraction methods. Its paramount advantage lies in the synergistic action of enzymes that selectively hydrolyze structural barriers and chemically bound forms, thereby liberating conjugated or entrapped bioactives while preserving their structural integrity, stereochemical configuration, and bioactivity.

#### 4.3.1. Phytosterols

Phytosterols (such as β-sitosterol, stigmasterol, and campesterol) are essential components of plant cell membranes and exhibit biological activities like cholesterol-lowering and anti-inflammatory effects. Traditional extraction methods are limited by the strong hydrophobicity of sterols and their predominantly esterified bound forms, where 60–85% exist as sterol esters, resulting in low yields and high energy consumption [108]. AEE significantly enhances the simultaneous extraction efficiency of both free sterols and sterol esters by targeting enzymatic hydrolysis of ester bonds combined with cell wall disruption technology. Cellulase/pectinase enzymes are used to degrade cell wall polysaccharides, releasing membrane-bound sterol-lipid complexes [109]. Lignin-degrading enzymes target woody plants to break down lignin-sterol cross-linked structures. In the extraction from soybean oil deodorizer distillate, immobilized lipase hydrolyzes sterol esters, achieving a total sterol recovery rate of 96%, while simultaneously yielding high-purity vitamin E with a recovery rate of 90% [110]. Laccase pretreatment coupled with esterase hydrolysis increased the yield of bound sterols from waste lignocellulose from 28% to 86%, providing a high-value utilization pathway for waste materials [111].

#### 4.3.2. Tocopherol

Tocopherols are predominantly distributed in plant oil bodies and seed endosperm, exhibiting high affinity for triglycerides. During oil extraction, they are readily released along with the lipid phase, resulting in relatively high solvent extraction efficiency [112]. However, the high temperatures involved in solvent extraction tend to cause oxidative degradation of tocopherols, particularly α-tocopherol. The core mechanism enabling the aqueous enzymatic extraction method to achieve high retention of tocopherols lies in its synergistic action effectively blocking degradation pathways. Mild enzymatic conditions significantly reduce the risk of thermal oxidation. The aqueous phase can accommodate chelating agents and water-soluble antioxidants, which synergistically scavenge free radicals and reactive oxygen species (ROS), thereby constructing an antioxidant protection network [113]. This effective blockade of degradation pathways, combined with its high release efficiency, collectively establishes the distinct advantage of the aqueous enzymatic method for obtaining oils enriched with high-activity γ-tocopherol. In practical application during rice bran oil production, replacing the traditional deodorization step with the aqueous enzymatic method achieved 88% tocopherol recovery rate, >90% tocopherol activity retention, and 50% reduction in energy consumption. Furthermore, employing composite enzyme treatment (protease + pectinase) yielded 91.5% tocopherol recovery rate and simultaneous production of high-purity soy peptides with an 85% yield [100,114].

#### 4.3.3. Carotenoid

Carotenoids are predominantly located within plastids or lipid droplets, often tightly bound to proteins or lipid complexes. Traditional organic solvent extraction requires disrupting cell walls and complex structures, resulting in cumbersome procedures and limited yields. In contrast, aqueous enzymatic extraction not only significantly enhances carotenoid extraction efficiency but also establishes a low-temperature, light-protected environment. This approach leverages the physical barrier effect of the aqueous phase and the synergistic protection of endogenous/exogenous antioxidants to block thermal degradation and detrimental isomerization pathways [115]. Consequently, it maintains high native molecular integrity and an ideal cis-trans isomer ratio. For instance, in tomato processing residues, combined pectinase and cellulase treatment increases carotenoid yield by 40–60% compared to traditional pressing [116]. Similarly, in *Haematococcus pluvialis*, cell wall disruption using snailase coupled with emulsification separation boosts astaxanthin extraction efficiency by 35% [117]. Given the thermal sensitivity of carotenoids, which suffer high loss rates and severe isomerization during conventional solvent extraction and refining, the aqueous enzymatic method achieves exceptional retention—preserving over 90% of the native cis-isomer configuration for compounds like β-carotene in the final product while delivering vivid and stable coloration [118].

#### 4.3.4. Polyphenols

AEE significantly enhances both the release efficiency and activity retention of polyphenols through targeted enzymatic cell wall disruption, demonstrating exceptional efficacy particularly for bound polyphenols where conventional methods fall short. While traditional solvent extraction efficiently isolates free polyphenols, it proves inadequate for extracting bound polyphenols, which constitute the majority of plant polyphenols and its reliance on high temperatures and harsh chemical treatments often induces oxidative polymerization and activity loss [119]. In contrast, the aqueous enzymatic approach achieves superior results by enzymatically degrading cell walls, thus hydrolyzing the cellulose–hemicellulose matrix to liberate encapsulated polyphenol-polysaccharide complexes while also cleaving the ester bonds of phenolic acids to release bound polyphenols [120]. Concurrently, the aqueous system eliminates organic solvent use, allowing direct recovery of polyphenol-enriched fractions via centrifugation while minimizing heat exposure. For instance, in grape seed processing, combined cellulase and pectinase treatment achieves a 92% proanthocyanidin yield, surpassing the 75% obtained by organic solvent methods, while improving the degree of polymerization retention by 30% [121]. Similarly, in wheat bran studies, a feruloyl esterase from *Eupenicillium parvum* combined with xylanase released about 72% of alkali-extractable ferulic acid, and pretreatment with dilute phosphoric acid further increased the yield to nearly 85%, while avoiding the structural degradation typically associated with alkaline hydrolysis [122].

As a sustainable extraction strategy, aqueous enzymatic extraction achieves efficient co-extraction of lipophilic bioactive compounds from oil-bearing matrices. This technology not only enhances extraction efficiency but also preserves the structural integrity and functionality of active constituents through its unique protective mechanisms, thereby significantly improving the oxidative stability, nutritional value, and sensory quality of oils. Tocopherols terminate free radical chain reactions via phenolic hydroxyl donation from their chromanol ring, while carotenoids quench singlet oxygen through energy transfer [123]. Polyphenols further amplify antioxidant effects by scavenging residual radicals, chelating pro-oxidant metal ions (Fe^2+^/Cu^2+^), and regenerating oxidized tocopherols. Phytosterols indirectly enhance stability by inhibiting thermal polymerization of oils. Experimental validation demonstrates these synergistic benefits. In enzymatically extracted rice bran oil, the combined action of tocopherols and γ-oryzanol extended the oxidation induction period from 42 to 126 h in Schaal oven tests, with a 31% suppression of acrylamide formation during frying [124]. Similarly, in aqueous enzyme-extracted olive oil studies, polyphenols effectively scavenged frying-derived free radicals while carotenoids quenched singlet oxygen, delaying the time required to reach 20 meq/kg peroxide value from 18 to 53 days under accelerated storage conditions and reducing conjugated diene increase by 57% [125].

## 5. Multiphase Proteins

**Figure 4:**
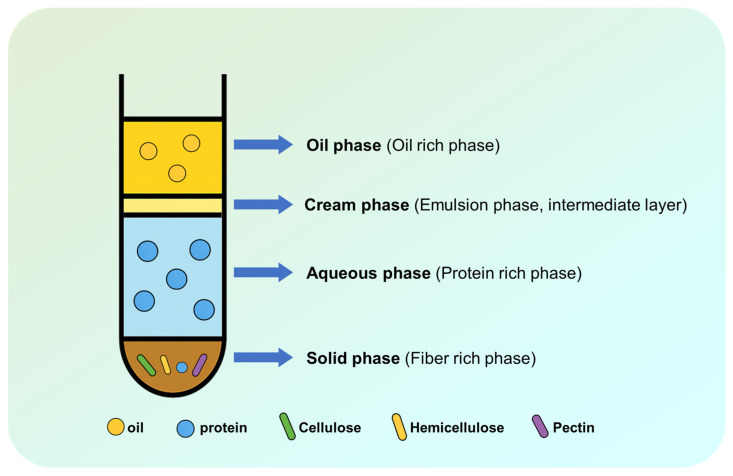
Three-phase distribution formed during aqueous enzymatic extraction.

During aqueous enzymatic extraction, the separation of the system into oil, emulsion, and aqueous phases represents a critical step that significantly influences the composition and properties of the resulting proteins (Figure 4). The distinct interfacial and physicochemical conditions within each phase, such as lipid–protein interactions, enzyme accessibility, and local molecular environment contribute to differences in protein structure, functionality, and recovery. This section discusses the characteristics of proteins distributed across these three phases, with emphasis on their structural features, functional properties, and potential applications based on phase-specific behaviors [126]. This provides a novel pathway for maximizing the retention-native quality of proteins and synergistically enhancing comprehensive resource utilization.

### 5.1. Aqueous Phase Protein

In AEE, the aqueous phase refers to the liquid fraction rich in soluble components after enzymatic hydrolysis. Aqueous phase proteins primarily consist of protein hydrolysates dissolved or dispersed in this phase, along with minor amounts of soluble native proteins. The structure of aqueous phase proteins is significantly altered by enzymatic hydrolysis, exhibiting highly hydrophilic and low-molecular-weight characteristics. As demonstrated by Xie et al., size-exclusion chromatography (SEC) of soybean meal hydrolysates revealed a strong shift toward low-molecular-weight peptides: the <1 kDa fraction rose from 42.14% to 69.65% as the degree of hydrolysis increased (6.76–22.02%), evidencing extensive proteolysis relative to native glycinin and β-conglycinin [127]. To put this into perspective, Native β-conglycinin is a trimer of roughly 150–200 kDa and glycinin is a hexamer of ~300–380 kDa, highlighting the drastic reduction in peptide size after hydrolysis [128]. Beyond molecular weight changes, Enzymatic hydrolysis selectively exposes hydrophilic amino acid residues on the protein surface. Hydrophobic residues either decrease in proportion within the peptides or become buried internally, resulting in markedly enhanced overall hydrophilicity [129]. The study by Li et al. demonstrated that enzymatic hydrolysis, particularly when combined with high-pressure homogenization, markedly modified the surface characteristics of peanut protein. The surface hydrophobicity (H_0_) decreased by approximately 50%, while hydrolysis significantly increased the exposure of hydrophilic and charged residues, thereby improving solubility and interfacial functionality [130]. Furthermore, Enzymatic hydrolysis induces a fundamental reorganization of protein secondary structure. This is robustly supported by the circular dichroism (CD) analysis of rice bran proteins reported by Wang et al. (2022), who observed that tryptic hydrolysis markedly decreased the α-helix and β-sheet contents while increasing β-turn and random-coil structures, indicating extensive unfolding and loss of ordered conformation [131]. This marked transition to a predominantly random coil state is a direct consequence of the extensive disruption of the native folded architecture during hydrolysis.

As summarized in Table 3, the distinct compositions and properties of aqueous phase proteins from various oilseeds render them highly promising for applications in health foods and pharmaceuticals, owing to their high solubility, rapid absorption, and diverse bioactivities. The core advantage of these proteins lies in the high bioavailability of their small-molecular-weight peptide components. Specifically, their absorption rate exceeds that of intact proteins by over 60%, enabling direct uptake via the intestinal peptide transporter system (PEPT1) within 30 min. This characteristic facilitates rapid delivery of amino acids required for post-exercise muscle damage repair, thereby enhancing anabolic efficiency [132]. Concurrently, it allows aqueous phase proteins to meet the demand for zero digestive burden protein supplementation among postoperative patients, the elderly, and individuals with digestive impairments, significantly improving nitrogen balance in these populations. Furthermore, the enzymatic release of functional peptides positions water-phase proteins as potent bioactive carriers for natural functional foods. For instance, their richness in ACE-inhibitory peptides enables blood pressure reduction through angiotensin-converting enzyme blockade [133]. Additionally, components containing sulfhydryl peptides and aromatic amino acid peptides demonstrate free radical scavenging and lipid oxidation retardation capabilities. These properties render water-phase proteins suitable for development into daily-consumption health beverages and anti-aging oral formulations.

### 5.2. Emulsion Phase Proteins

In AEE, the emulsion phase represents a key product fraction, composed primarily of lipids, interfacially adsorbed proteins/peptides, phospholipids, and other amphiphilic compounds. Emulsion phase proteins, defined as proteins or peptide fragments adsorbed at the oil–water interface, undergo structural alterations induced by enzymatic hydrolysis, interfacial adsorption, and environmental stresses [145]. Owing to their unique amphiphilic structure and exceptional emulsifying functionality, emulsion phase proteins serve as the critical factor enabling efficient oil recovery in AEE. Moreover, they exhibit significant potential as natural emulsifiers and sources of functional ingredients. Unlike the intact proteins present in the raw material, emulsion phase proteins typically result from partial proteolytic hydrolysis.

The defining characteristic of emulsion phase proteins is their significantly enhanced amphiphilicity. Although these proteins exhibit a broad molecular weight distribution, they are generally smaller than their native counterparts. This reduced molecular size (0.1–10 µm) facilitates rapid diffusion and adsorption onto oil–water interfaces. Chutinara et al. (2024) reported that pepsin-hydrolyzed lentil proteins exhibited much smaller hydrodynamic diameters (~100 nm aggregates) measured by dynamic light scattering, resulting in faster adsorption at oil–water interfaces compared with the native protein [146]. According to the Stokes–Einstein relation (D ∝ 1/R_h_), this reduction in particle size implies a higher diffusion coefficient, thereby facilitating the rapid interfacial migration and assembly of hydrolysates during emulsification [146]. During enzymatic hydrolysis, selective cleavage of peptide bonds exposes hydrophobic amino acid residues that were originally buried within the protein core. Concurrently, peptide segments containing hydrophilic residues are retained [147]. Zhang et al. (2023) combined fluorescence-based surface hydrophobicity assays with LC–MS/MS peptide profiling to show that controlled hydrolysis of soybean protein isolate by a cell envelope proteinase unfolded the molecules, increased surface hydrophobicity, and generated peptide fractions enriched in hydrophobic amino acids (e.g., Leu-rich sequences), while more polar segments remained in the soluble phase [148]. This coexistence of hydrophobic and hydrophilic domains transforms them into highly efficient amphiphilic molecules capable of orienting themselves at oil–water interfaces. Furthermore, enzymatic hydrolysis disrupts the protein’s rigid structure, increasing peptide chain flexibility [149]. Onsaard et al. (2022) deconvoluted the FTIR amide I band and showed that native rice bran protein contained about 15.6% α-helix and 41.3% β-sheet, whereas enzymatic hydrolysates exhibited reduced α-helix together with higher β-sheet and random-coil fractions, indicating disruption of ordered structures and a more flexible, disordered conformation that enables rapid conformational adjustment at interfaces [150]. Upon adsorption, the hydrophobic regions of these peptides embed into the oil phase while hydrophilic regions extend into the aqueous phase, often in cooperation with endogenous phospholipids and other surface-active components released from the raw materials, so that a composite protein–phospholipid interfacial layer is formed which synergistically stabilizes the emulsion system [151].

In aqueous enzymatic extraction, emulsion phase proteins are recovered by demulsification via methods such as isoelectric precipitation and heat treatment. The resulting aggregates are then separated by centrifugation, washed, and dried to yield the final product, showcasing significant application potential due to their exceptional natural emulsifying capacity and inherent bioactivity. Among these, their roles as natural emulsifiers/stabilizers in the food industry and as green functional ingredients in cosmetics represent two of the most prominent application avenues. The advantage of emulsion phase proteins lies in the amphiphilic peptides generated through enzymatic hydrolysis. These peptides rapidly adsorb at oil–water interfaces, forming robust interfacial films [152]. This unique property positions them as an ideal alternative to synthetic emulsifiers or high cost animal derived ingredients. In plant-based dairy analogues, they effectively stabilize fat globules, preventing phase separation and enhancing sensory smoothness [32]. For comminuted meat products, they inhibit fat separation during processing and storage, thereby improving textural juiciness. In sauces and dressings, they facilitate the formation of stable emulsified systems while simultaneously aligning with clean-label trends. Furthermore, the natural surface activity and biocompatibility of milk phase proteins pave the way for their utilization in cosmetics. As primary emulsifiers in creams and lotions, they stabilize both oil-in-water (O/W) and water-in-oil (W/O) systems, potentially replacing a portion of synthetic surfactants to reduce irritation risks for sensitive skin [153]. Concurrently, their constituent antioxidant peptides may synergistically enhance the efficacy of anti-aging formulations, while the inherent moisturizing properties of small peptides render them suitable for incorporation into serums and hydrating lotions.

### 5.3. Solid Phase Proteins

During AEE, the solid phase primarily consists of undissolved proteins along with residual cell wall materials, partially undigested starch, minerals, and minor bound lipids. The most defining structural characteristic of solid phase proteins is their pronounced insolubility, as they remain undissolved or undispersed under enzymatic hydrolysis and aqueous conditions. Wali et al. (2017) reported that power—ultrasound pretreatment of rapeseed protein prior to enzymatic hydrolysis increased the surface hydrophobicity of the resulting hydrolysates by about 130%, which they attributed to the exposure of hydrophobic groups and which promoted stronger hydrophobic associations and the formation of larger aggregates [154]. Recent work on insoluble soy peptide aggregates likewise shows that the precipitated fraction formed during protease hydrolysis is stabilized by multiple intermolecular forces: Jiang et al. (2024) used reductive dissolution to demonstrate that hydrophobic interactions, hydrogen bonding and disulfide cross-links all contribute to the poor solubility of these aggregates, while Yang et al. (2023) found that disulfide bonds and hydrogen bonds are the dominant forces reinforcing heat-induced gels from soybean protein hydrolysates [155,156]. These covalent and non-covalent interactions, together with ionic interactions, collectively drive the formation of a densely cross-linked, insoluble protein network. Compared to native proteins or soluble peptides in the aqueous phase, solid-phase proteins also undergo marked changes in secondary structure. FTIR analysis during aqueous enzymatic extraction of rapeseed oil showed that protein secondary structure gradually shifted from more ordered α-helix/β-sheet motifs toward disordered structures as hydrolysis progressed, consistent with protein unfolding, denaturation and aggregation in the solid residue [157]. Furthermore, solid-phase proteins are typically tightly bound to or embedded within partially degraded cell-wall polysaccharides; this physical entrapment, combined with intensive intermolecular interactions, severely restricts their solubility.

Despite its limited solubility and functional properties, the solid phase protein residue generated from aqueous enzymatic extraction holds significant potential for diversified applications, owing to its high protein content, abundant dietary fiber, superior water- and oil-binding capacities, and well-preserved nutritional profile [158]. With typically elevated protein levels in dry matter, this residue serves as a high-quality plant-derived protein source, providing essential amino acids, minerals, and vitamins [159]. As a low-cost processing byproduct, it can be used directly or in dried/powdered form to partially replace conventional protein meals in livestock and aquaculture feeds. Functionally, rich in insoluble dietary fiber, the solid-phase proteins function as a high-fiber additive in baked goods to boost fiber content; A water/oil-retaining filler in meat products (e.g., sausages, patties), substituting starch or soy protein while enhancing moisture retention and a texturizing agent in plant-based meat analogues to improve fibrous mouthfeel.

## 6. Conclusions and Future Prospects

Aqueous enzymatic extraction (AEE) has demonstrated unique advantages as a green biomanufacturing strategy for oils and proteins. By precisely deconstructing cell walls and oil bodies under mild aqueous conditions, AEE enables efficient lipid release, preserves fatty acid integrity, and significantly enriches thermally sensitive bioactive compounds. In addition, its ability to recover multiphase proteins—including aqueous-, emulsion-, and solid-phase fractions—broadens the scope of resource utilization and highlights the potential of AEE to move oilseed processing beyond single-product extraction toward integrated high-value valorization. The integration of physical assistance technologies such as ultrasound, microwave, ultra-high pressure, and pulsed electric fields further enhances mass transfer, suppresses emulsification, and improves functional quality, thereby advancing AEE toward industrial feasibility.

Despite these benefits, several challenges still constrain the large-scale application of AEE. Enzyme costs remain relatively high, demulsification efficiency in complex systems is insufficient, and the structural and functional roles of emulsion- and solid-phase proteins are underexplored compared with aqueous-phase proteins. To unlock the full potential of AEE, future research should focus on the rational design of tailored enzyme cocktails, coupling with advanced multiphase separation and valorization technologies, and developing intensified and continuous industrial processes. A deeper understanding of protein structure–function relationships across different phases will also be essential. With these advances, AEE can evolve into a scalable, low-carbon, and economically viable technology, providing a robust platform for sustainable utilization of oils and proteins in food, pharmaceutical, and bio-based industries.

## Figures and Tables

**Table 1:** Effects of key process parameters in aqueous enzymatic extraction.

Process Parameter	Impact on Oil Extraction Yield	Impact on Protein Quality	Impact on Energy Consumption & Economics	Reference
Enzyme Type	Combined enzymes (cellulase + pectinase) yield > 90%, significantly higher than single enzyme (~70%); proteases increase free oil yield by 5–15% via demulsification.	Proteases lead to a degree of hydrolysis (DH) of 5–15%; excessive hydrolysis (DH > 15%) increases bitter peptides and reduces the emulsifying activity index by over 30%.	Combined enzymes cost 20–50% more than single enzymes, but overall economics can be optimized via higher yield and reduced downstream demulsification steps.	[15,32,33,34,35]
Enzyme Concentration	Oil yield increases significantly within 1.0–2.5%range (40–85%); plateaus with minimal gains (<5% increase) above 3.0%.	Concentrations > 2.5% cause over-hydrolysis, reducing emulsifying stability by 20–40% with negligible solubility improvement (<5%).	Enzymes account for 30–60% of production cost; each 0.5% concentration increase raises cost by 12–18%	[12,36,37,38]
Reaction Time	Oil yield peaks (85–92%) within 60–90 min; extending to 120 min increases yield <3%, but may raise oil peroxide value by 0.5–1.0 meq/kg	Time > 120 min reduces protein emulsifying capacity by 25% with no significant solubility gain, increasing microbial risk.	Each 30 min extension increases energy use by 15–25%; optimal timing saves 20–35% in total energy.	[29,36,39,40]
Reaction Temperature	Maximum oil yield (85–90%) at 45–55 °C; >60 °C causes rapid enzyme deactivation, reducing yield by 20–30%; <40 °C reduces reaction rate by 50%.	Temperatures > 65 °C induce protein denaturation, reducing Nitrogen Solubility Index (NSI) by 15–25%; <40 °C lowers protein yield by 30–40%.	Temperature control consumes 40–60% of total energy; each 5 °C increase raises energy use by 12–15%; optimal range maximizes energy efficiency.	[41,42,43,44]

**Table 3:** AEE-Derived Aqueous Phase Proteins across Oilseeds.

Raw Material	Type & Molecular Weight	Key Functional Property	Application Potential	References
Soybean	AEE skim proteins & hydrolysates; peak < 10 kDa; UF 3–5 kDa	Higher solubility; fewer antinutritional factors	Nutrition supplements; acidic-pH beverages	[134,135,136]
Rice bran	Soluble proteins/hydrolysates; UF < 3, 3–5, 5–10 kDa; ≤3–5 kDa most active	Antioxidant and ACE-inhibitory; emulsification improved	Functional foods and beverages	[137,138]
Rapeseed/Canola	AEE skim proteins/hydrolysates; low-MW enriched (peak < 10 kDa); UF 3–5 kDa	High solubility; strong emulsifying/interfacial film-forming	Clean-label emulsifiers; plant-based beverages/creams	[139,140,141]
Peanut	Skim and demulsified interfacial proteins; low-MW enriched (peak < 10 kDa); UF 3–5 kDa	Superior interfacial/emulsifying performance; umami-enhancing peptides identified	Plant-based beverages/creams; clean-label emulsifiers	[142,143,144]

## Data Availability

The original contributions presented in the study are included in the article. Further inquiries can be directed at the corresponding author.
